# A case–control study on the association of intestinal flora with ulcerative colitis

**DOI:** 10.1186/s13568-021-01267-9

**Published:** 2021-07-15

**Authors:** Yin-hua Tang, Hong-cheng Liu, Guang Song, Tian-tian Wu, Ying Zhao, Li-jun Shi

**Affiliations:** 1 Department of Gastroenterology, The First Affiliated Hospital, Harbin Medical University, No. 23, Youzheng Street, Nangang District, Harbin, 150001 Heilongjiang China; 2 Department of Digestive internal medicine, Xiang’an Hospital of Xiamen University, No. 2000, Xiang’an East Road, Xiang’an District, Xiamen, 361101 China; 3 Department of Infectious Diseases, Kaifeng Central Hospital, No. 85, Hehe Street, Longting District, Kaifeng City, China; 4 Department of Digestive internal medicine, Chifeng Municipal Hospital, Chifeng Clinical Medical School of Inner Mongolia Medical University, No. 1 Zhaowuda Road, Hongshan District, Chifeng City, China

**Keywords:** Ulcerative colitis, Intestinal flora, Species diversity, Species richness

## Abstract

The association between intestinal flora and ulcerative colitis (UC) was studied in order to provide a basis and method for clinical treatment. Fresh fecal samples were collected from 30 active UC patients and 10 healthy controls. The intestinal flora DNA from each sample was extracted and 16S rRNA gene sequencing was carried out using HiSeq platform to identify the intestinal flora in fecal samples. The richness and diversity of intestinal flora in UC patients were significantly lower than those in healthy control group (P < 0.05). Significant differences were observed between the intestinal flora-species of UC patients and healthy controls. *Synergistetes* (P < 0.01) and *Firmicutes* (P < 0.05), along with probiotics *Veillonella* (P < 0.01), *Ruminococcus* and *Coprococcus* (P < 0.05) in the UC patients were lower than that in the healthy controls significantly. Furthermore, compared with the control group, *Tenericutes* (P < 0.01) and intestinal pathogenic bacteria, including *Bacteroides* (P < 0.01), *Escherichia* and *Sutterella* (P < 0.05) were significantly increased. The incidence of UC is significantly associated with the changes in intestinal flora. Changes in intestinal flora may lead to a decrease in the diversity of intestinal flora or to the enrichment of a particular intestinal flora.

**Supplementary Information:**

The online version contains supplementary material available at 10.1186/s13568-021-01267-9.

## Introduction

Ulcerative colitis (UC) is a chronic non-specific intestinal inflammatory disease of unknown etiology. Its main symptoms include abdominal pain, diarrhea and mucus, pus and blood. Some UC patients have extra-intestinal manifestations and complications, which affect their daily lives, and even their health (Whitehead 2016; Sykora et al. 2018). An epidemiological survey reported higher prevalence of UC among the people of 20–40 years, and higher prevalence in males than females (Lewin et al. 2019). The incidence rate of UC fluctuates from 5.5 to 24.3 per million people worldwide with significant regional and ethnic specificity. Statistically, the prevalence of UC in China is about 11.6 per million (Whitesides 1985; Molodecky et al. 2012; Ng et al. 2013; Cui et al. 2018). The pathogenesis of UC has been reported to be associated with environmental, genetic, immune and intestinal flora-factors (Cui et al. 2018). Among them, the barrier, metabolic and nutritive function of intestinal flora, immune system function, and the regulation of the balance of intestinal flora play a key role in the pathogenesis of UC.

The intestinal flora is currently considered to be a complex ecosystem, which is (Ahlawat et al. 2021b) composed of 500–1000 different species, accounting for 10^14^ bacterial cells, which is 10 times more than that of the total number of human cells (Domingo and Sanchez 2018). The genome of all intestinal microorganisms is known as “microbiome”, which is more than 100-times larger than the human nuclear genome (Huang et al. 2019). Intestinal microflora is increasingly recognized as a key factor of human health. Several studies have shown that many human chronic diseases are associated with intestinal microecological disorders (Tripathi et al. 2018; Zhang et al. 2018; Ma et al. 2019; Ahlawat et al. 2021a). The intestinal flora is an important environmental factor, which has been reported to be associated with a series of metabolic diseases, including obesity (Machiels et al. 2014) and diabetes (Yuan et al. 2018). It is crucial for human beings to maintain a good symbiotic relationship between human body and intestinal flora.

Although the specific pathogenesis of UC remains unclear, the interactions of genetic abnormalities, immune system dysfunction, intestinal barrier dysfunction and microbiological infection are thought to be the early risk factors of UC (Deng et al. 2019; Lewin et al. 2019; Lee et al. 2020). The microbiological factor has been recognized as the most potent environmental factor in the UC progression, which is possibly associated with the harmful mucosal invasion, activation of carcinogens, or inflammatory responses (Zheng et al. 2019). Moreover, the inflammatory cytokines and/or mixed inflammatory infiltrates in the intestinal mucosa may also be responsible for the development of UC (Kopecki et al. 2019). Therefore, a better understanding of the gastrointestinal microflora and inflammatory cytokines in the progression of UC has been of great significance.

This study provides a new basis and method for the clinical assessment of UC by analyzing the association between changes in intestinal flora and UC. It is of great importance to fully understand the changes of intestinal flora in UC. This study provides a theoretical basis for the regulation of intestinal flora in the treatment of UC and its complications.

## Materials and methods

We have uploaded the data to the NCBI Sequence Read Archive database (Accession Number: PRJNA695366).

### Materials

Agencourt AMPure XP magnetic beads and Elution Buffer were purchased from MGI Tech Co., Ltd. InhibitEX Buffer and Qubit^®^ dsDNA BR Assay Kit were purchased from Shanghai Haoran Biotechnology Co., Ltd. Two wash buffers, including Buffer AW1 and Buffer AW2 were purchased from Shanghai Beinuo Biotechnology Co., Ltd. Buffer AL and Buffer ATE were purchased from Shanghai Limin Industrial Co., Ltd. KAPA HiFi HotStart DNA Polymerase was purchased from Shanghai HiFi Biotech Co., Ltd.

### Sample collections and setting participants

According to the revised standards of “Consensus Opinions on Diagnosis and Treatment of Inflammatory Bowel Disease” formulated by the Inflammatory Bowel Disease Group of the Chinese Medical Association Gastroenterology Branch in 2018, 30 patients with ulcerative colitis were recruited from the First Affiliated Hospital, Harbin Medical University from January to December 2019. There were no gender restrictions. The inclusion criteria for the recruitment of patients included: (1) region, Heilongjiang province; (2) age, 30–65 years; (3) past history of ulcerative colitis, the ulcerative colitis active period; and (4) the patients are not treated with antibiotics and microecological agents or not treated with these drugs 4 weeks before the onset. The core standards included: (a) hematology tests, such as hemoglobin (Hb), white blood cells (WBC), erythrocyte sedimentation rate (ESR) and C-reactive protein (CRP); (b) colonoscopy report showing ulcerative colitis; and (c) pathology report showing moderate to severe mucosal chronic inflammation or suggesting crypt abscess. The exclusion criteria of the UC patients included: (a) incomplete clinical data; (b) patients with severe heart, liver, lung, and kidney diseases; (c) patients with diabetes and severe infections; (d) patients with pregnancy and lactation; (e) patients with UC complications; (f) patients with extra-intestinal manifestations; and (g) patients with other autoimmune diseases. In addition, 10 healthy controls in the physical examination center of the First Affiliated Hospital, Harbin Medical University were recruited, regardless of gender. For the inclusion criteria of the recruitment of healthy controls, the region, age, and drugs usage were same as that of UC patients. Other inclusion criteria parameters included: (1) no history of chronic gastrointestinal diseases and routine physical examination and stool examination showed no abnormalities; and (2) information collection such as hematological tests, such as Hb, WBC, ESR, and CRP. The sample collection was reviewed by the Ethics Committee of the First Affiliated Hospital of Harbin Medical University.

Fresh stool samples of 1–10 g were collected in a medical stool collection vessels (the surface of the vessels had sample numbers) from the patients and healthy controls, and stored in a − 80 °C low-temperature freezer after one hour of sampling.

### DNA extraction and sequencing

The intestinal floras from the fecal samples were homogenized and genomic DNA was extracted using QIAamp Fast DNA Stool Mini Kit (QIAGEN China (Shanghai) Co., Ltd.). The quality and integrity of the DNA extraction were evaluated using micro spectrophotometer and gel electrophoresis.

The extracted DNA samples and corresponding fusion primers were mixed in order to configure the PCR reaction system. The PCR reaction parameters were set for PCR amplification. Agencourt AMPure XP magnetic beads were used to purify the PCR amplification products, which were then dissolved in Elution Buffer, and labeled. The metagenomic library was prepared and the fragment range and concentration were analyzed using Agilent 2100 Bioanalyzer. The prepared colonies were selected for sequencing on HiSeq platform based on insert size.

### Data filtering

The original sequencing data was processed to remove low-quality, low-complexity, pollution and N-terminal sequences and obtain clean data. FLASH (Fast Length Adjustment of Short reads, v1.2.11) software was used to sequence paired-end sequences using overlapping relationships The obtained paired-end sequences were assembled into a sequence to obtain the tags of hyper-variable region. USEARCH (v7.0.1090) software was used to perform clustering and splicing at 97% similarity. The spliced tags were clustered as OUT (Operational Taxonomic Units) and compared with the database and species annotations. The statistics table of OTU abundance for each sample was obtained, and the samples were analyzed on the basis of OTU and species annotation results.

### Statistical analysis

Statistical analysis was carried out using Excel spreadsheets and SPSS statistical software. The differences in the species diversity and richness between the UC patients’ samples and that of healthy controls were analyzed. The rank sum test was used, and the results were analyzed using OTU Rank curve, alpha diversity box plot and beta diversity box plot. The similarity in the species of both the groups was analyzed by OTU PLS-DA (Partial Least Squares Discriminant Analysis) method. The RDP classifier Bayesian algorithm was used for species composition analysis and statistics. The histogram and heat map of species richness and the comparison histogram of key species difference were used for the analysis of species composition between two groups. *U* test was used to calculate the average relative abundance of species between the two groups and to analyze the significance of the difference test. The statistical differences of P < 0.05 and P < 0.01 were considered as significant and extremely significant, respectively. FastTree is used to construct evolutionary tree by selecting OTU or sequence corresponding to classification information at a certain level.

## Results

### Comparison of species diversity and richness

**Fig. 1:**
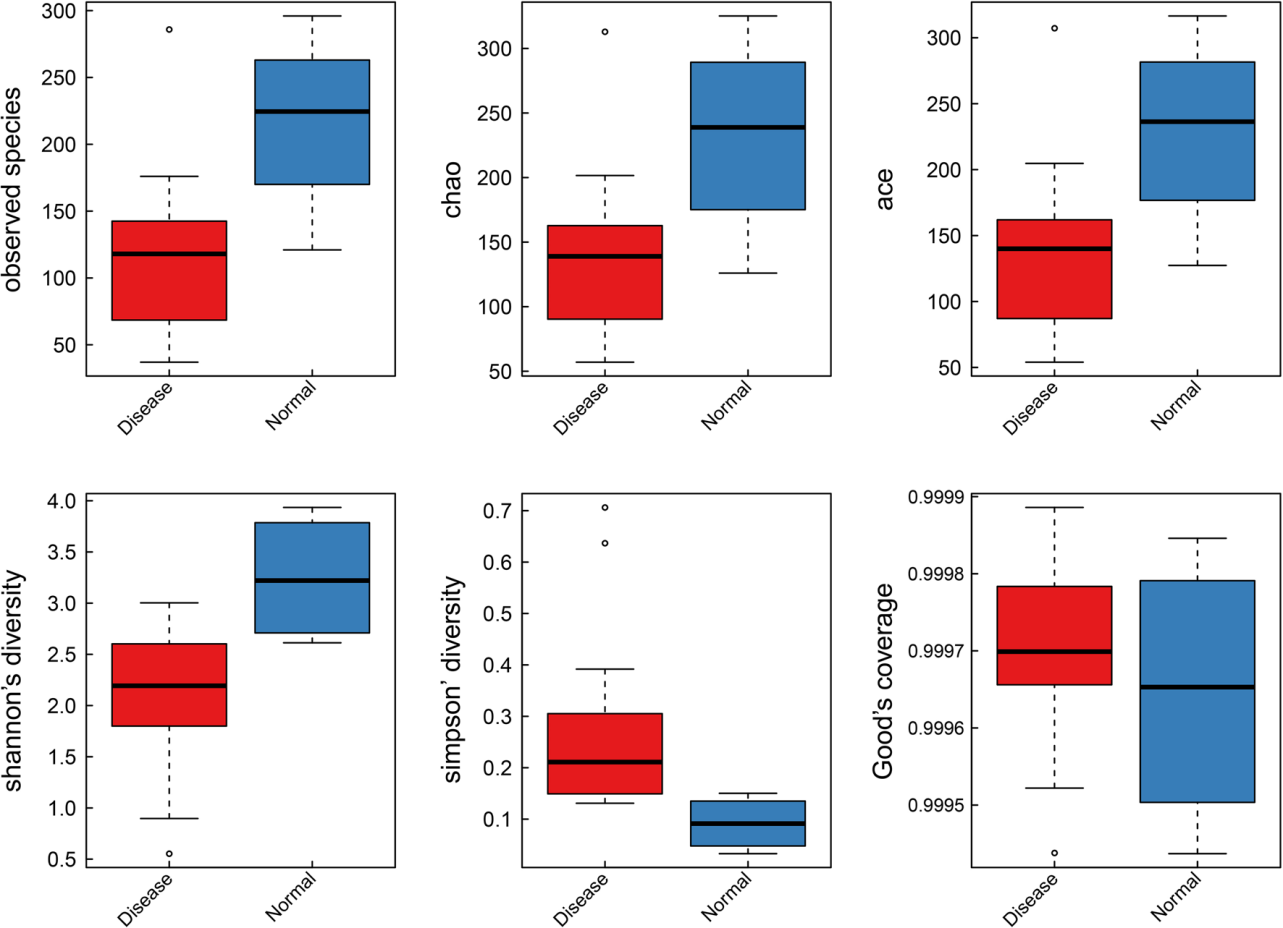
Comparison of Alpha diversity box between disease group (UC patients) and normal group (healthy controls). Red and blue colored boxes show the richness of microflora in disease and control samples, respectively for all the statistical analysis mentioned with each graph. Clear significant differences were observed in all the statistical analyses

**Fig. 2:**
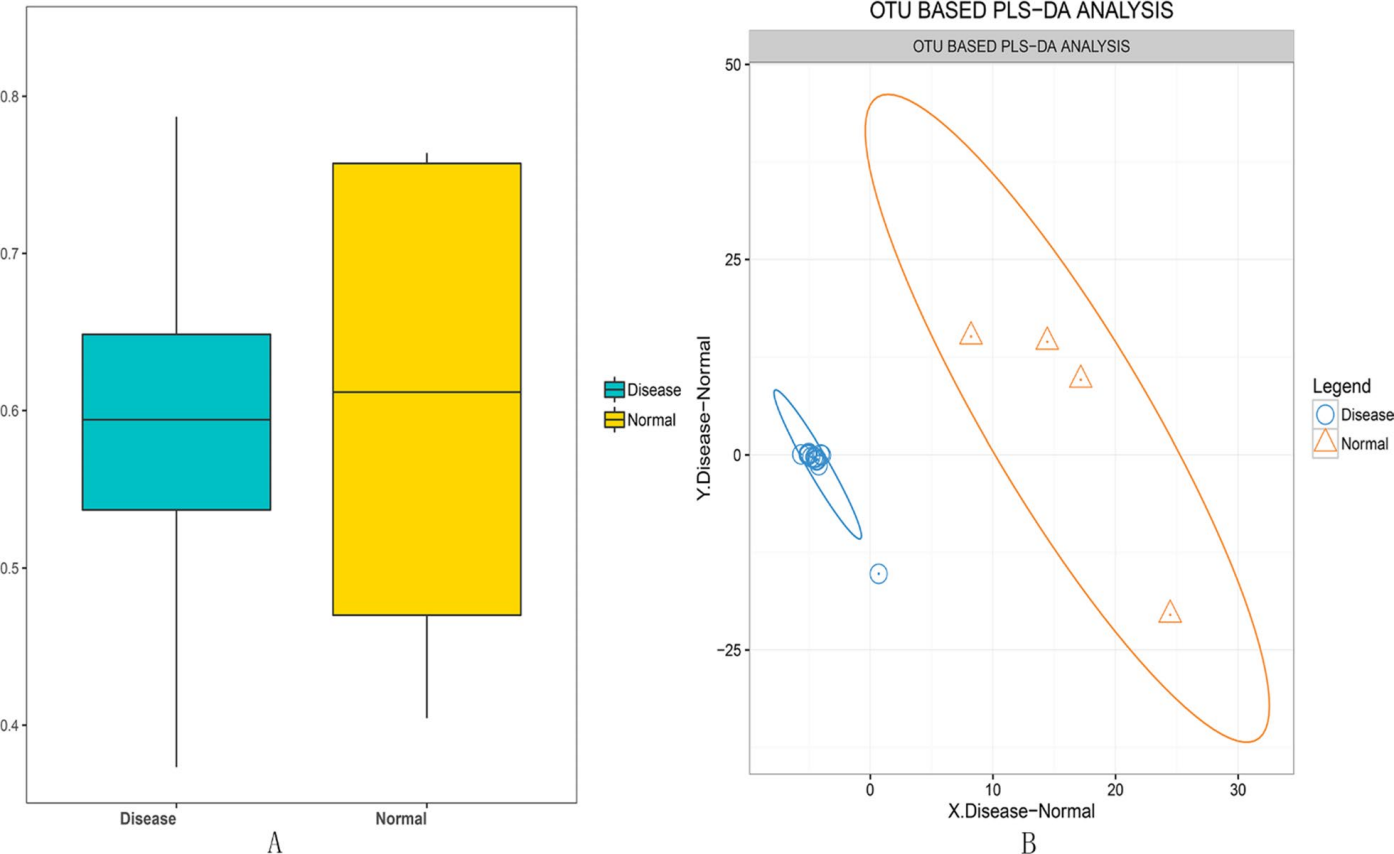
Unweighted UniFrac Beta diversity box and the histograms of species richness at phylum level, showing species diversity between UC patients and healthy controls (normal). **A** Cyan and yellow colored boxes show the diversity of microflora in disease and control samples. The species in the control samples were more diverse as compared to those in the disease samples. **B** Color identification of each phylum is shown in the side bar. The disease samples shows greater number of *Bacteriodetes* and *Proteobacteria* and lesser number of *Firmicutes* and *Verrucomicrobia* than the normal samples

**Fig. 3:**
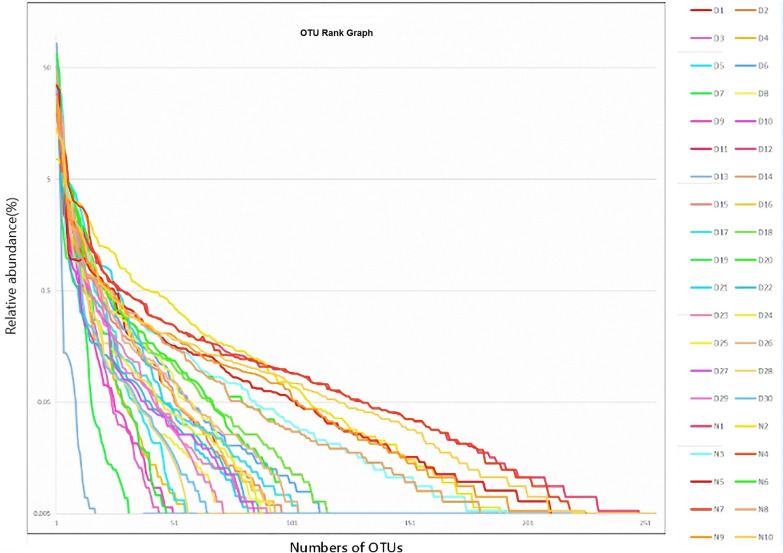
OTU Rank graph showing the comparison of species richness between UC patients and healthy controls (normal). The side bar shows color identifications for 30 disease samples and 10 normal samples, denoted as D1-30 and N1-10, respectively. A clear divergence in the normal samples OTUs was observed

The comparison of species diversity between the UC patients and the healthy controls is shown in Figs. 1, 2, 3. Shannon index, simpson index and unweighted UniFrac Beta diversity box plot were used to reflect the diversity of bacterial community in the samples (Fig. 1). The species diversity in the UC patients was significantly lower than that in the health controls (P < 0.05). The comparison of species richness between the UC patients and the healthy controls is shown in Figs. 1, 3. The observed species index, chao index, and ACE index reflected the community richness between the two groups of samples. The species richness in the UC patients was significantly lower than that of the healthy controls (P < 0.05). The biodiversity statistics between the two groups are listed in Table 1.

**Table 1:** Statistics of biodiversity index in UC patients and healthy controls

	Mean (disease)	SD (disease)	Mean (normal)	SD (normal)	*P*
Observed species	114.2	63.54211	216.5	72.17802	0.04019
Shannon index	2.0797	0.69312	3.24729	0.63804	0.00929
Chao index	134.87980	66.44779	232.23235	82.53096	0.0485
Ace index	134.85776	65.65546	229.1388	78.12063	0.0485
Simpson index	0.27035	0.17975	0.09145	0.05329	0.00619

### Comparison of species similarity

The species similarities between the samples of the UC patients and healthy controls were analyzed on the basis of two sets of OTU data and OTU PLS-DA analysis chart. As shown in Fig. 2B, the intestinal flora clustering of the UC patients and healthy controls showed distinct distribution from each other, without showing any intersection, which showed significant differences in the species diversity between the two sets of samples.

### Species level composition analysis

**Fig. 4:**
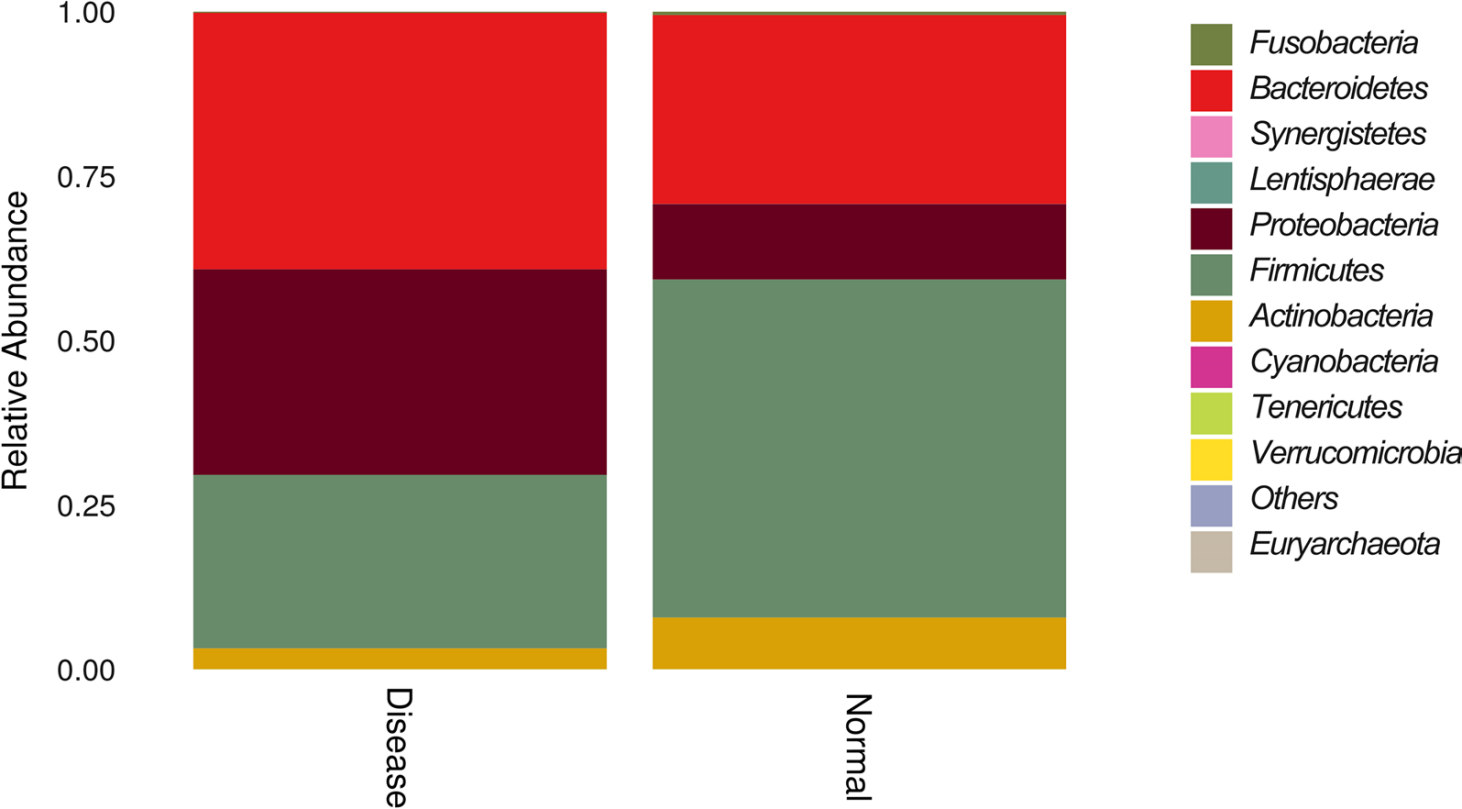
Comparison of the histograms of species richness at phylum level between UC patients and healthy controls (normal). Color identification of each phylum is shown in the side bar. The disease samples shows greater number of *Bacteriodetes* and *Proteobacteria* and lesser number of *Firmicutes* and *Verrucomicrobia* than the normal samples

**Fig. 5:**
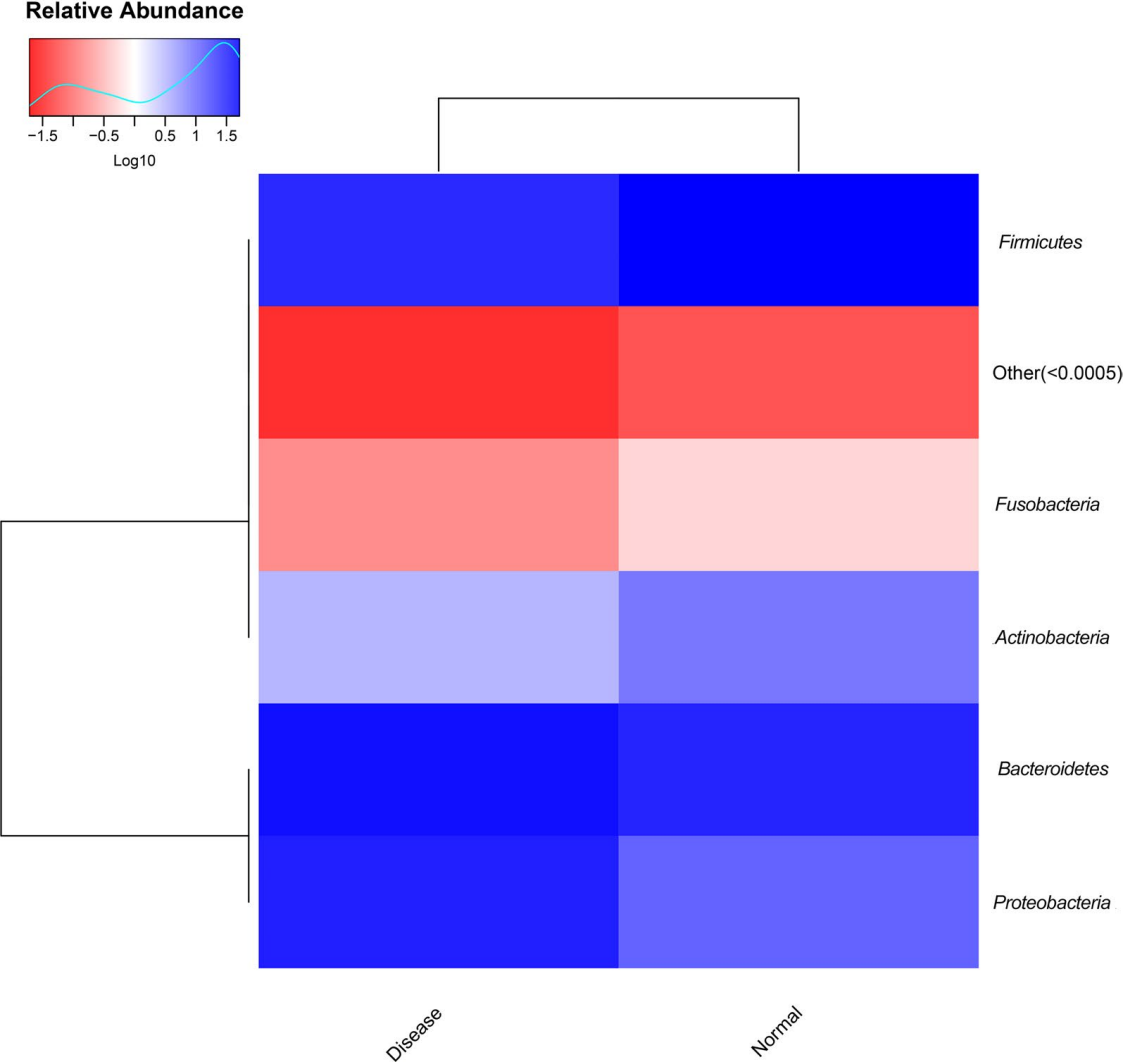
Heat map showing horizontal species richness between UC patients and healthy controls (normal). The relative abundance of bacteria is shown in terms of color intensities (denoted by the scale given in upper left corner). The differences in the color intensities between disease and normal samples show differences in the number of respective species

**Fig. 6:**
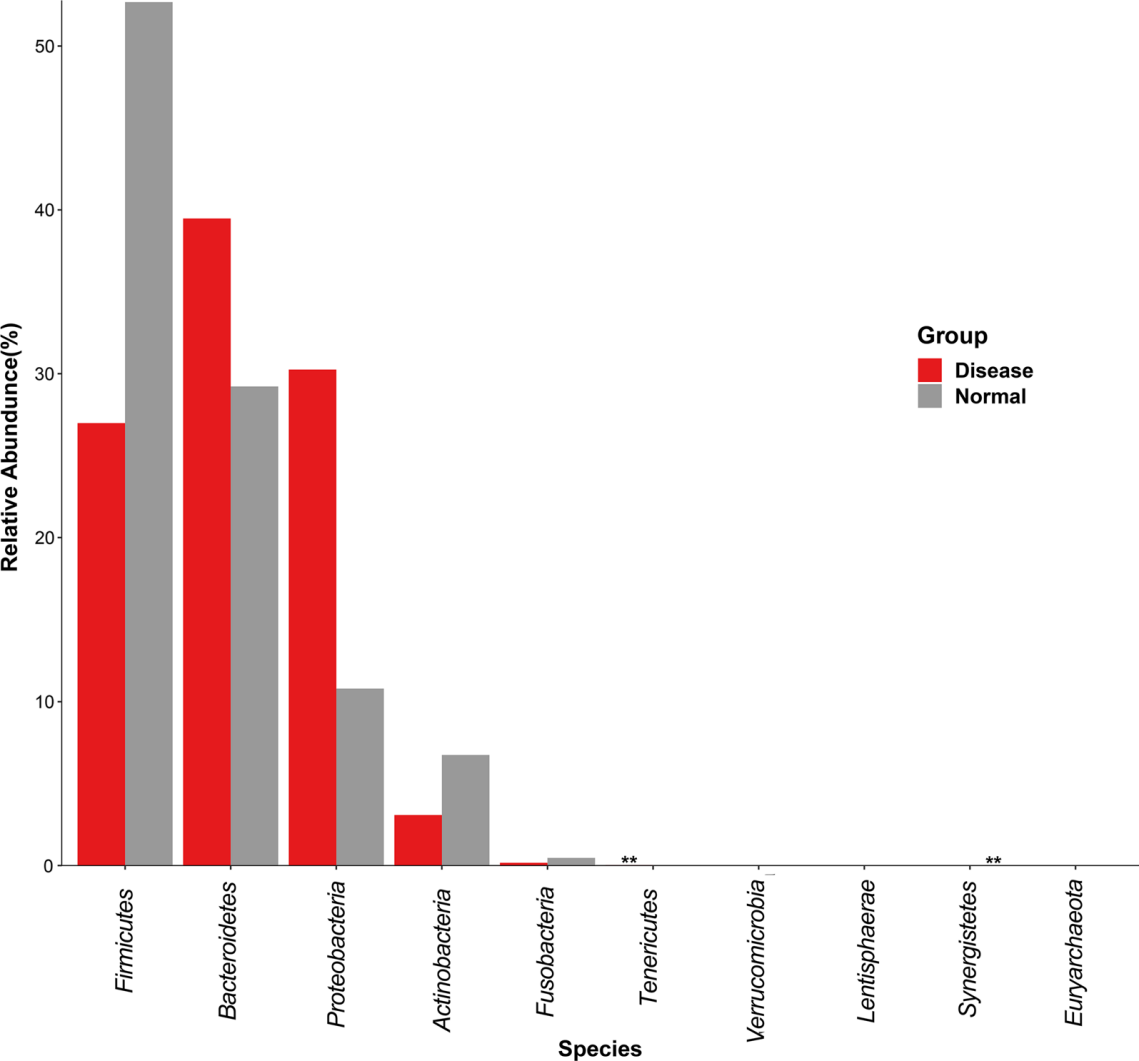
Histogram of key species showing differences between species in UC patients and healthy controls (normal). The species in disease and normal samples are denoted by red and grey bars, respectively, where the length of bars denotes the relative abundance of species

The histogram and heat map of the species richness of the UC patients and healthy controls are shown in Figs. 4, 5, respectively. The average relative abundance and the significance of difference test are shown in Fig. 6 and provided in Additional file 1: Table S1. The probabilities of species distribution of the two groups are listed in Table 2. *Synergistetes* and *Firmicutes* were decreased in the UC patients as compared to the healthy control with statistical significance (P < 0.01 and P < 0.05), respectively. Compared with healthy controls, patients with UC showed a significant increase in *Tenericutes* (P < 0.01). There were no significant differences in *Bacteroides*, *Proteobacteria*, *Actinomycetes*, *Fusobacteria*, *Verrucomicrobia*, *Lentisphaerae*, *Euryarchaeota* between the UC patients and healthy controls.

**Table 2:** Species distribution of samples at phylum level in UC patients and healthy controls

	Disease	Normal
*Fusobacteria*	0.001728302	0.005181347
*Bacteroidetes*	0.39010419	0.287266
*Synergistetes*	1.21E−05	0.000122781
*Lentisphaerae*	0	0.000139152
*Proteobacteria*	******	0.11469358
*Firmicutes*	0.263940147	0.513567272
*Actinobacteria*	0.031726043	********
*Cyanobacteria*	1.21E−05	0
*Tenericutes*	*******	0.00021282
*Verrucomicrobia*	8.74E−05	0.000130966
Others	7.03E−06	9.41E−05
*Euryarchaeota*	0	4.09E−05

### Genus level composition analysis

**Fig. 7:**
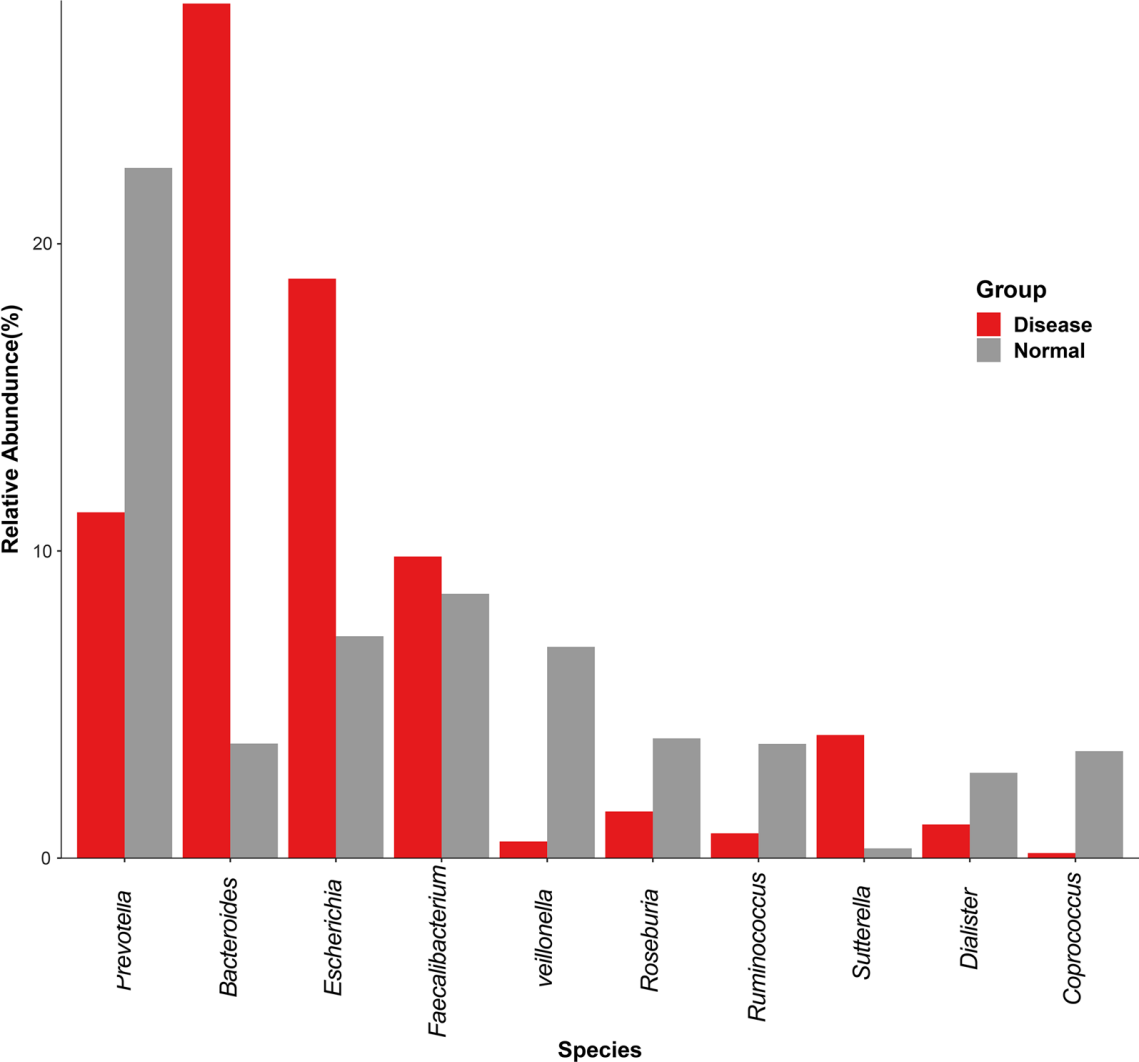
Histogram showing bacterial species composition in UC patients and healthy controls (normal). The species in disease and normal samples are denoted by red and grey bars, respectively, where the length of bars denotes the relative abundance of species

The average relative abundance of species in the UC patients and healthy controls is shown in Fig. 7 and provided in Additional file 1: Table S2. The beneficial bacteria *Veillonella* were significantly reduced in the UC patients as compared to the healthy controls, along with significant increase in harmful bacteria *Bacteroides* in the UC patients as compared to the healthy controls (P < 0.01). *Firmicutes* were also reduced in the UC patients as compared to the healthy controls with statistical significance of P < 0.05. The differences in *Bacteroides*, *Proteobacteria*, *Actinomycetes*, *Fusobacteria*, *Verrucomicrobia*, *Lentisphaerae*, *Euryarchaeota* between the UC patients and healthy controls were insignificant.

### Phylogenetic analysis of species

**Fig. 8:**
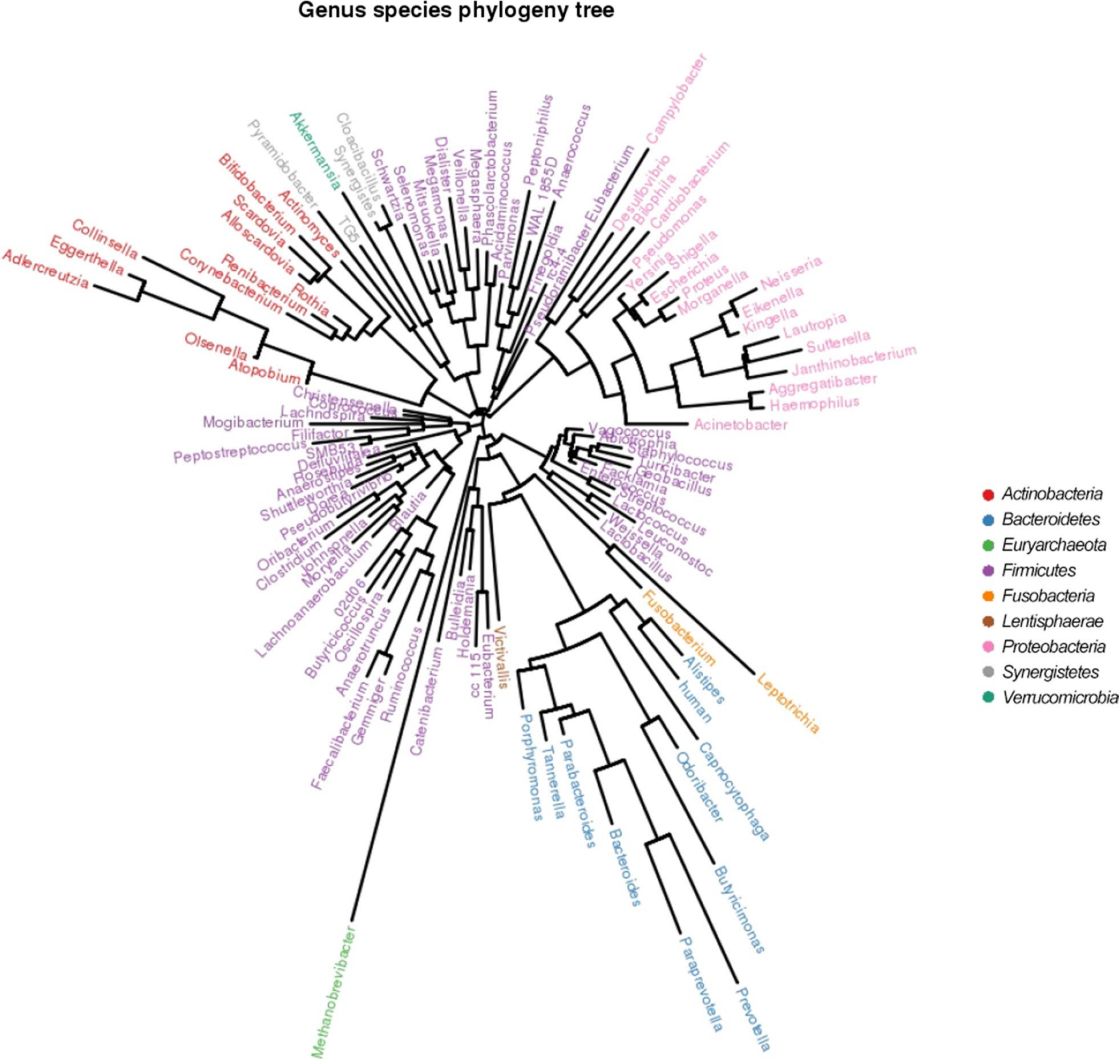
The phylogenetic analysis of species level

The phylogenetic analysis of species level was studied as follows. *Firmicutes* and *Proteobacteria* were the two largest intestinal microbial species, which also the fastest growing species. Second, *Actinobacteria* and *Bacteroidetes* have more branches than other species. *Euryarchaeota, Fusobacteria, Lentisphaerae,* and *Verrucomicrobia* have less species evolution (Fig. 8).

## Discussion

In this study, a case–control experimental method was adopted and 16S rRNA gene sequencing technology using Illumina HiSeq 2500 sequencing platform was applied to explore the relationship between changes in intestinal flora of UC patients and healthy controls. The results showed that the species richness and diversity of the intestinal flora in the UC patients were significantly lower than those of healthy controls. Consistent with the domestic and foreign studies, significant differences in the species diversity of the intestinal flora between UC patients and healthy controls were observed (Ruili et al. 2012; Min et al. 2015). It has been reported that the UC recurrence in the patients in UC remission period is associated with the aggravation of intestinal flora imbalance. It also found that the status of intestinal flora of UC patients is closely associated with the development of the disease by testing intestinal flora in the stool samples of UC active period, UC remission period and healthy controls (Min et al. 2015). Another study have also revealed that the diversity of intestinal flora in patients with active UC is lower than that in UC remission and healthy controls, analyzed by ERIC-PCR (enterobacterial repetitive intergenic consensus PCR) technology (Ruili et al. 2012).

It was found that the *synergistetes* and *firmicutes* decreased significantly, and the Soft-walled bacteria significantly increased in the UC patients as compared to healthy controls. The current research showed that the alterations in the intestinal flora of UC patients were mainly attributable to decrease in *Firmicutes* and increase in *Proteobacteria*. The trend of changes in *Actinobacteria* and *Bacteroides* has not been confirmed yet (Hold et al. 2014). Following these differences, the differences in the composition of intestinal flora in UC patients and healthy controls at genus level were analyzed, which showed that *Escherichia* and *Sutterella* were significantly increased in UC patients. The increase in *Escherichia* has been reported to disrupt the balance and integrity of intestinal environment and promote inflammation. A study reported that *Helicobacter* is a major flora in intestinal infections (Li et al. 2017). When the distribution of the flora changes, it can cause biliary infections, urinary tract infections, and intra-abdominal infections, and even life-threatening. *Sutterella* sp. may cause digestive disorders (Sartor and Wu 2017), which may be another factor that affect the aggravation and complications of UC patients. *Coprococcus,* a major flora found in the intestines of humans and animals, decreased significantly in the intestinal flora of UC patients, which can produce natural antibiotics, enhance intestinal function, and also benefit the body. *Coprococcus,* not only produce bacteriostatic substances, such as bacteriocins, that inhibit the growth of pathogenic bacteria, but they can also inhibit the reproduction of urease-producing bacteria and spoilage bacteria in the intestinal tract. Through these mechanisms it improves the protection of the intestinal microenvironment and reduces endotoxin and inflammation. *Ruminococcus* are the main fiber-degrading bacteria in gastrointestinal tract, which produce succinic acid, acetic acid, formic acid, ethanol and lactic acid, and build the intestinal microbial barrier alongside with other flora.

Our research showed a higher number of *Bacteroides* in UC patients than the healthy controls, which was consistent with a previous study (Furusawa et al. 2013). In our present study, the number of *Faecalibacterium* genus was relatively high as compared to the healthy controls, which was inconsistent with previous studies. One of the representative species of *Clostridium prausnitzii* is *Faecalibacterium prausnitzii*. *Clostridium prausnitzii* is a major probiotic in the gastrointestinal tract. It plays vital role in the formation of intestinal mucosal barrier, reduction of intestinal inflammation, competitive inhibition of pathogenic bacteria and reduction of pathogenic bacterial colonization. An early study showed that the number of *Clostridium prasmoides* in UC patients was significantly lower than healthy controls (Machiels et al. 2014). Man et al. (Man et al. 2013) compared the number of *Bacteroides*, *Clostridium tenuiens* and *Bifidobacteria* in UC patients and healthy controls. They showed an increased number of *Bacteroides* in UC patients, which could promote inflammation, and a decreased number of *Clostridium tenuis* and *Bifidobacterium,* which could inhibit inflammation (Man et al. 2013). We speculated that the UC patients who participated in this study did not take medication within 4 weeks. The accuracy of this experiment on the effect of medication was not studied in this study. The long course of UC patients, repeated symptoms and long-term use of aminosalicylic acid, biological agents, glucocorticoids, microecological agents, etc. might have impact on the results of this study. The degree of inflammation in UC patients is also an important factor that affect intestinal flora. This study did not strictly distinguish among patients having mild, moderate, and severe UC. Therefore, the role of these factors still needs to be studied in more detail. In addition, the age, gender, and living habits of the study participants can lead to different research conclusions. This requires the sample size to be expanded and refined and the sample variables to be specified as much as possible.

This study explored the feasibility of using intestinal flora to guide the treatment of UC by studying the intestinal flora of UC patients. Probiotics, such as *Clostridium prasylvia*, can positively regulate the stability of intestinal flora by increasing the permeability of intestinal mucosa and producing butyrate to reduce intestinal inflammation (Jinli et al. 2019); *Lactobacillus* regulates the balance of intestinal flora by promoting digestion and inhibiting the growth of potential pathogenic bacteria. *Faecococcus* can decompose nutrients to produce lactic acid, enhance intestinal function and inhibit the growth and reproduction of pathogenic bacteria. Fecal bacteria transplantation is another application of intestinal flora. By transplanting the intestinal flora of healthy people, it can reshape the intestinal flora barrier and can treat intestinal diseases. At present, there is evidence that the intestinal probiotics and intestinal fecal bacteria transplantation have a certain effect on the treatment of UC (Ding et al. 2019; Paramsothy et al. 2019). However, the therapeutic application of intestinal flora also has certain risks, especially in the immune-compromised people (such as patients with long-term use of glucocorticoids or immune-suppressants, patients with immunodeficiency, etc.). Therefore, the application of intestinal flora treatment for such people should be carefully considered. There may be risks of bacterial migration and the transmission of drug-resistant factors (Haiying et al. 2008). Thus, the extensive applications of intestinal flora still need a lot of clinical investigations.

## Supplementary Information

**Additional file 1: Table S1.** Average relative abundance and significance of difference test of key species. **Table S2.** Composition of bacterial species in disease group and normal group.

## Data Availability

All data generated or analysed during this study are included in this published article and its Additional file 1.

## Abbreviations

UC: Ulcerative colitis
Hb: Hemoglobin
WBC: White blood cells
ESR: Erythrocyte sedimentation rate
CRP: C-reactive protein
