# Supplementary material for: A case–control study on the association of intestinal flora with ulcerative colitis
Source: AMB Express. 2021 Jul 15;11:106. doi: 10.1186/s13568-021-01267-9 (PMC8282830; doi:10.1186/s13568-021-01267-9)
Supplement: Supplementary file 1 — Additional file 1: Table S1. Average relative abundance and significance of difference test of key species. Table S2. Composition of bacterial species in disease group and normal group. [file 13568_2021_1267_MOESM1_ESM.docx]

**Additional file 1**

**Table S1. Average relative abundance and significance of difference test of key species**

| Phylum | Mean (Disease) | SD (Disease) | Mean (Normal) | SD (Normal) | *P* | FDR |
| --- | --- | --- | --- | --- | --- | --- |
| *Actinobacteria* | 3.081742 | 7.260533 | 6.748712 | 12.887805 | 0.9613 | 0.9613 |
| *Bacteroidetes* | 39.473475 | 24.327799 | 29.22323 | 17.15574 | 0.410733 | 0.448072 |
| *Firmicutes* | ***** | 15.739553 | 52.682643 | 23.797283 | 0.048504 | 0.121202 |
| *Proteobacteria* | 30.251331 | ******* | 10.802222 | ******* | 0.152219 | 0.228328 |
| *Fusobacteria* | 0.167076 | 0.433844 | ******* | 0.557847 | 0.212466 | 0.283288 |
| *Synergistetes* | 0.001261 | ********* | 0.012907 | ******** | 0.002943 | 0.035316 |
| *Tenericutes* | 0.032426 | 0.125587 | 0.022275 | 0.023035 | 0.009497 | 0.056982 |
| *Verrucomicrobia* | 0.009313 | ******** | 0.014382 | ******* | 0.060288 | 0.121202 |
| *Lentisphaerae* | 0 | 0 | 0.014967 | 0.029934 | ******** | 0.121202 |
| *Euryarchaeota* | 0 | 0 | 0.004402 | 0.008804 | 0.070701 | 0.121202 |

**Table S2. Composition of bacterial species in disease group and normal group**

| Genus | mean(Disease) | SD(Disease) | mean(Normal) | SD(Normal) | *P* |
| --- | --- | --- | --- | --- | --- |
| *Prevotella* | 11.258149 | 23.654289 | 22.470785 | 17.988775 | 0.078794 |
| *Bacteroides* | ******8 | 24.16023 | 3.728363 | 4.175701 | 0.0084727 |
| *Escherichia* | 18.865707 | 27.583031 | 7.223175 | ******* | 0.0424355 |
| *Faecalibacterium* | 9.817703 | 13.534909 | 8.604923 | 9.544335 | 0.726223 |
| *Dialister* | 1.094485 | 2.457164 | 2.777633 | 4.460898 | 0.170308 |
| *Ruminococcus* | 0.807056 | 0.718005 | ******** | 4.825139 | 0.0228088 |
| *Coprococcus* | ******** | 0.199641 | 3.482337 | 3.964853 | 0.034219 |
| *Sutterella* | 4.007515 | 6.607593 | 0.316931 | ****** | 0.029287 |
| *Roseburia* | 1.518591 | ******** | 3.899045 | 5.527319 | 0.291592 |
| *Veillonella* | 0.541427 | 0.888487 | 6.878568 | ******* | 0.00530444 |
